# Supplementary material for: Astrocytic ACSBG1 depletion improves lipid-cytokine signaling and attenuates α-Synuclein pathology in a Parkinson’s disease mouse model
Source: bioRxiv. 2026 Jun 8:2026.05.20.726454. Originally published 2026 May 21. Preprint. [Version 2] doi: 10.64898/2026.05.20.726454 (PMC13228550; doi:10.64898/2026.05.20.726454)
Supplement: Supplementary file 1 [file media-1.pdf]

Supplementary Information

Supplementary file 1. DEG data from RNA-seq, on primary astrocyte of WT and *Acsbg1* KO, treated with PBS, or TNF-α / IL-1α (excel file)

Supplementary file 2. Lipid LC-MS data from conditioned medium, from primary astrocyte of WT and *Acsbg1* KO, treated with PBS, or TNF-α / IL-1α (excel file)

Supplementary table 1. Materials used for this study

Reagent or resource	Source	Identifier
**Antibodies**		
Anti-Alpha-synuclein antibody (clone 42)	BD	RRID: AB_398107
p-S129 Synuclein (clone EP1536Y)	Abcam	RRID: AB_869973
p-S129 Synuclein (clone D1R1R)	Cell Signaling Technology	RRID: AB_279886
Anti-Vinculin	Sigma-Aldrich	RRID: AB_477629
Anti-GFAP	Novus Biologicals	RRID: AB_829022
Anti-Iba1	Wako Chemicals	RRID: AB_839504
Anti-Tyrosine Hydroxylase (TH)	EMD Millipore	RRID: AB_390204
Anti-Acsbg1	Atlas Antibodies	RRID:AB_2677593
Alexa Fluor® 488 AffiniPure Donkey Anti-Mouse IgG (H+L)	Jackson ImmunoResearch	RRID: AB_2340846
Donkey anti-Rabbit IgG (H+L) Highly Cross-Adsorbed Secondary Antibody, Alexa Fluor™ 555	Thermo Fisher Scientific	RRID: AB_162543
Donkey anti-Goat IgG (H+L) Cross-Adsorbed Secondary Antibody, Alexa Fluor™ 488	Thermo Fisher Scientific	RRID: AB_2534102
**Cytokine and lipids**		
TNF-alpha Recombinant Protein	Sigma-Aldrich	Catalog# SRP3177
IL-1alpha Recombinant Protein	Sigma-Aldrich	Catalog# SRP3310
Human CCL5 (RANTES) Recombinant Protein	Thermo Fisher Scientific	Catalog# 300-06
Human CCL20 (MIP-3 alpha) Recombinant Protein	Thermo Fisher Scientific	Catalog# 300-29
Oleic Acid	Sigma-Aldrich	Catalog# O3008
Sphingosine (C16:1, C18:1, C20:1, C22;1)	From the Lab	-
**Kit**		
VECTASTAIN(R) ELITE(R) ABC anti-Rabbit IgG HRP Immunodetection Kit	Vector Laboratories	Catalog #PK-6101
Mouse Cytokine Antibody Array (Membrane, 62 Targets)	abcam	Catalog # ab133995
Papain dissociation kit	Worthington Biochemical	Catalog # LK003178
**Experimental models: Organisms/strain**		
Mouse: wild type (WT: C57BL/6J)	Jackson Laboratory	Catalog #000664
Mouse: *Thy1-α-Syn TG Line 61*	Jackson Laboratory	RRID: IMSR_JAX:038796
Mouse: *Acsbg1 KO*	Jackson Laboratory	RRID: IMSR_JAX:024754
**Plasmid DNA**		
pcDNA3.1-Acsbg1-(k)DYK	Genescript	Catalogue: OHu06859
pcDNA3.1-Acsbg1^{K701A}-(k)DYK	From the Lab	-
**Oligonucleotides**		

Genotyping primer for WT sequence of *Acsbg1 KO* forward: anneal at 58 degrees<br>5'- TCCACAGACAGGTGACTAA -3'	Sigma-Aldrich	N/A
Genotyping primer for WT and *Acsbg1 KO* common reverse: anneal at 58 degrees<br>5'- CACTGAACATGGTTTGAGC -3'	Sigma-Aldrich	N/A
Genotyping primer for KO sequence of *Acsbg1 KO* forward: anneals at 58 degrees<br>5'- AGTACTGTGGTTTCCCAAATGTGTCA -3'	Sigma-Aldrich	N/A
Genotyping primer for Thy1-α-Syn TG reverse: anneals at 57 degrees<br>5'- GATGATGGCATGCAGCACTGG -3'	Sigma-Aldrich	N/A
Genotyping primer for Thy1-α-Syn TG reverse: anneals at 57 degrees<br>5'- GACGGGTGTGACAGCAGTAGC -3'	Sigma-Aldrich	N/A
Cloning forward primer for Acsbg1^{K701A} mutant: anneals at 58 degrees<br>5'- tcccacgatggcgctgaaacggc -3'	Sigma-Aldrich	N/A
Cloning reverse primer for Acsbg1^{K701A} mutant: anneals at 58 degrees<br>5'- cccaactctccaccc -3'	Sigma-Aldrich	N/A

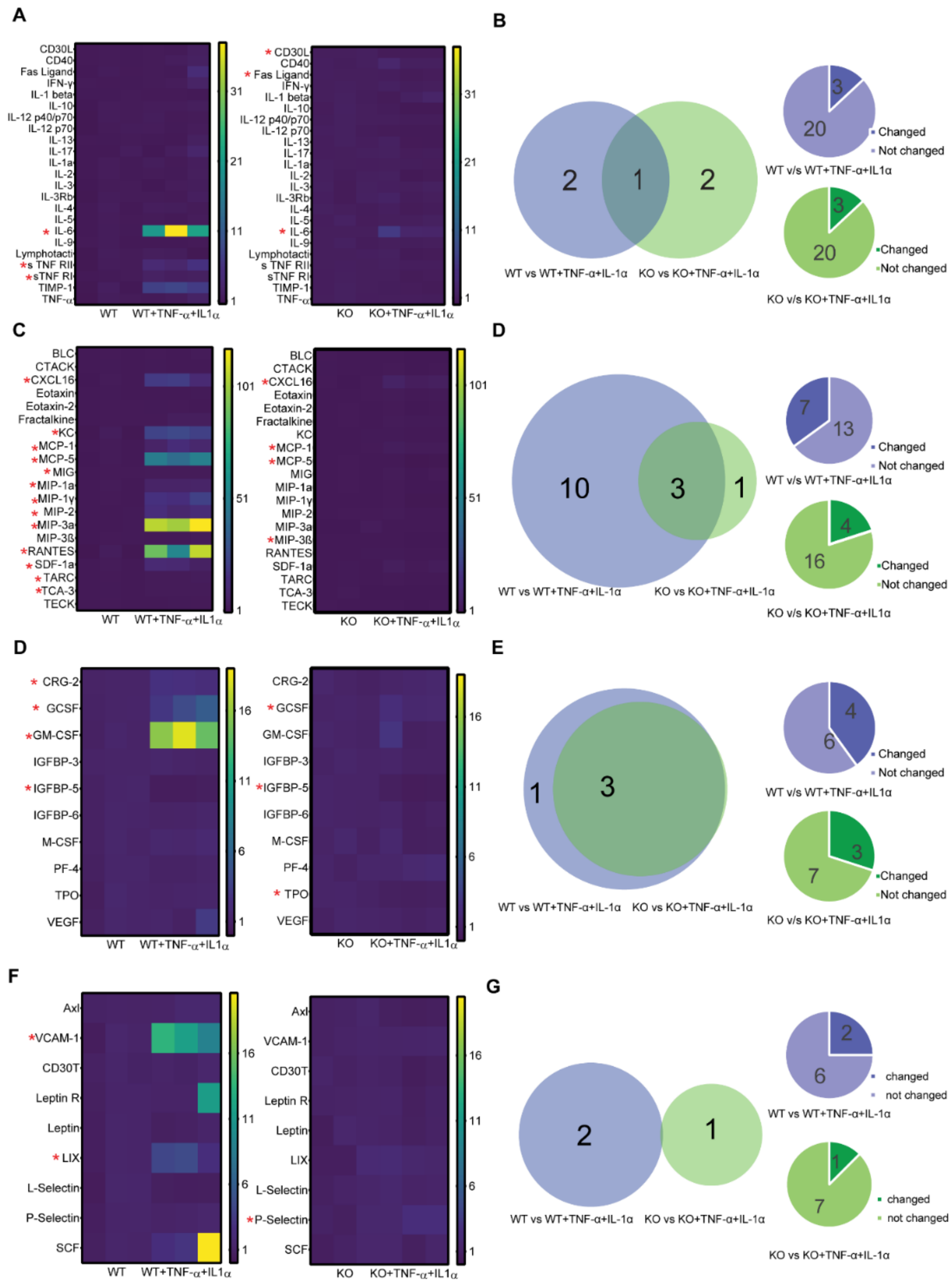

Supplementary Figure 1. Conditioned media from *Acsbg1* KO show reduced inflammatory ligands secretion from *Acsbg1* KO astrocytes

(**A, C, D, F**) Heatmaps depicting the levels of cytokines (**A**), chemokines (**C**), growth factors (**D**), and other inflammatory mediators (**F**) measured in conditioned media from wild-type (WT) or Acsbg1 knockout (KO) astrocytes under basal conditions (PBS-treated, naive) or following pro-inflammatory stimulation with TNF-α and IL-1α (pathologically reactive state). Color scale represents relative expression intensity. Analytes marked with a red asterisk (*) indicate statistically significant differences. n=3 per group; *$p<0.05$.

(**B, D, E, G**) Venn diagrams (**left panels**) illustrating the overlap in significantly changed inflammatory mediators between WT vs. WT+TNF-α+IL-1α (blue) and KO vs. KO+TNF-α+IL-1α (green) comparisons. Pie charts (**right panels**) show the number of cytokines were significantly changed (colored) versus unchanged (light) in WT vs. WT+TNF-α+IL-1α (blue, upper) and KO vs. KO+TNF-α+IL-1α (green, lower).

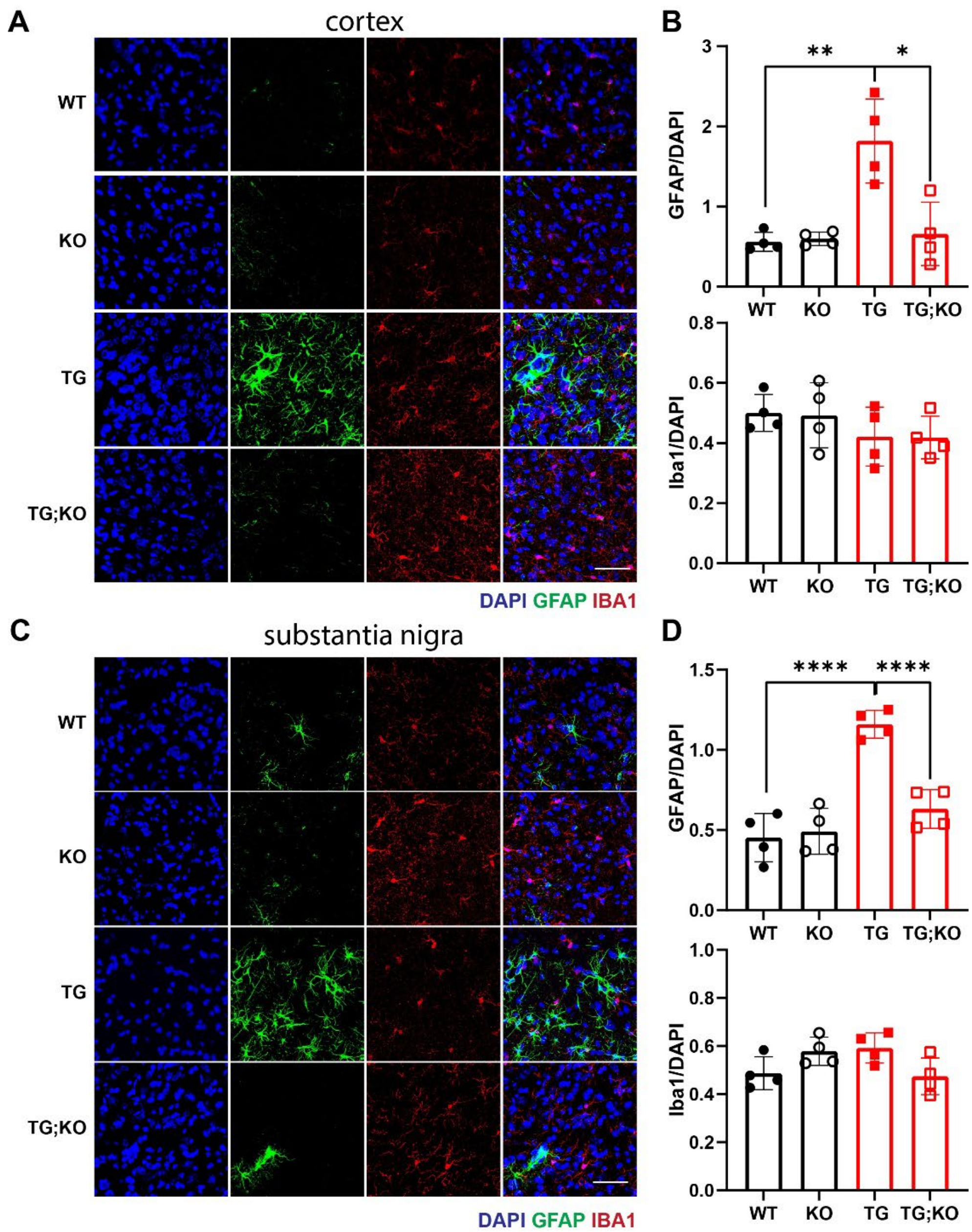

Supplementary Figure 2. ***Acsbg1*** **deletion reduces reactive astrogliosis but not microglial activation in Thy1-α-Syn transgenic mice.**

(A, C) Representative immunofluorescence images of brain sections stained for DAPI (blue), GFAP (green), and IBA1 (red) from WT, KO, TG, and TG;KO mice at cortex and substantia nigra. Scale bar, 50 µm. **(B)** Quantification of GFAP (top) and IBA1 (bottom) immunoreactivity normalized to DAPI in the regions shown in (A). p* < 0.05, p**<0.01, n = 4 per group. **(D)** Quantification of GFAP (top) and IBA1 (bottom) immunoreactivity normalized to DAPI in the regions shown in (C). **** p < 0.0001, n = 4 per group.

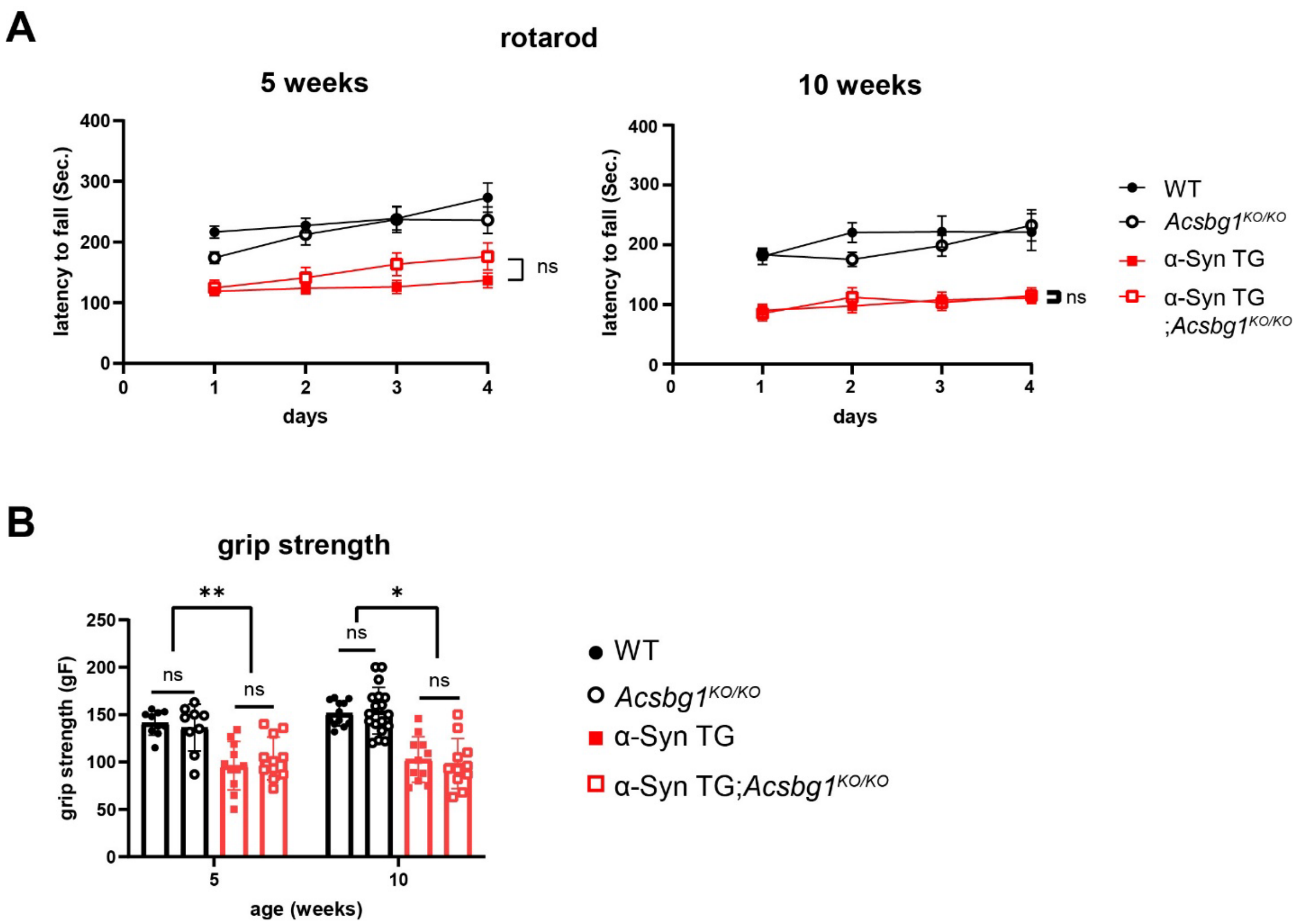

Supplementary Figure 3. Characterization of the motor function and weight difference of α-syn TG mice with *Acsbg1* KO background. We compared the motor function of Thy1-α-Syn TG (TG) and its *Acsbg1* KO background (TG;KO) to wild-type and *Acsbg1* KO littermates at 5 and 10 weeks of age. (**A**) motor coordination in the rotarod test, and (**B**) motor strength by grip strength test. * $p<0.05$, ** $p<0.01$, ns=non-significant. WT (n=7 for 5 weeks and 14 for 10 weeks), *Acsbg1$^{KO/KO}$* (n=15 for 5 weeks, n=16 for 10 weeks), Thy1-α-Syn TG (n=9 for 5 weeks, n=15 for 10 weeks), Thy1-α-Syn TG*; Acsbg1$^{KO/KO}$* (n=12 for 5 weeks, n=13 for 10 weeks). All mice used for the behavior test are male mice due to Thy1-α-syn TG construct being on the X chromosome.
